# Design of an Optimal Convolutional Neural Network Architecture for MRI Brain Tumor Classification by Exploiting Particle Swarm Optimization

**DOI:** 10.3390/jimaging11020031

**Published:** 2025-01-24

**Authors:** Sofia El Amoury, Youssef Smili, Youssef Fakhri

**Affiliations:** 1 Laboratory RI, Faculty of Sciences of Kenitra, Ibn Tofail University, Kenitra 14000, Morocco; fakhri@uit.ac.ma; 2 ENIC, Faculty of Science and Technology of Settat, Hassan First University, Settat 26000, Morocco

**Keywords:** convolutional neural networks (CNN), Particle Swarm Optimization (PSO), brain tumor image classification, optimal CNN architecture, medical image classification

## Abstract

The classification of brain tumors using MRI scans is critical for accurate diagnosis and effective treatment planning, though it poses significant challenges due to the complex and varied characteristics of tumors, including irregular shapes, diverse sizes, and subtle textural differences. Traditional convolutional neural network (CNN) models, whether handcrafted or pretrained, frequently fall short in capturing these intricate details comprehensively. To address this complexity, an automated approach employing Particle Swarm Optimization (PSO) has been applied to create a CNN architecture specifically adapted for MRI-based brain tumor classification. PSO systematically searches for an optimal configuration of architectural parameters—such as the types and numbers of layers, filter quantities and sizes, and neuron numbers in fully connected layers—with the objective of enhancing classification accuracy. This performance-driven method avoids the inefficiencies of manual design and iterative trial and error. Experimental results indicate that the PSO-optimized CNN achieves a classification accuracy of 99.19%, demonstrating significant potential for improving diagnostic precision in complex medical imaging applications and underscoring the value of automated architecture search in advancing critical healthcare technology.

## 1. Introduction

The rapid advancements in information and communication technologies, coupled with the increasing capabilities of computing resources, have enabled significant progress in the domains of artificial intelligence and machine learning. Among these advances, convolutional neural networks (CNN) have emerged as essential tools for the processing and classification of images [1]. Their ability to autonomously identify and extract salient features from visual data [2] makes them valuable allies in various fields, including medical image classification [3]. Beyond medical applications, CNN are widely used in areas such as autonomous driving [4], where they assist in object detection and scene segmentation; security [5], where they enhance facial recognition and surveillance systems; and in the field of agriculture, where they aid in crop and livestock monitoring by analyzing aerial images captured by drones [6]. These diverse applications demonstrate the versatility and significance of CNN in solving complex, real-world problems across multiple domains.

While CNN have shown great promise in various applications, they are often criticized for their “black box” nature, meaning their decision processes are not easily interpretable. This lack of transparency can be particularly problematic in critical domains such as medical imaging, where understanding the rationale behind a model’s decisions is essential for trust and safety. Moreover, CNN are vulnerable to adversarial attacks, where slight modifications to input data can lead to misclassification, posing significant risks in practical applications [7]. Addressing these challenges is crucial for enhancing the reliability and safety of CNN-based medical imaging systems.

Magnetic resonance imaging (MRI) is a powerful, non-invasive tool frequently used in diagnosing and monitoring brain tumors. Brain tumors refer to abnormal growths of cells within the brain that can be either benign or malignant, with various types such as gliomas, meningiomas, and pituitary tumors [8]. MRI produces high-resolution, detailed images of the brain, enabling precise visualization of tumor characteristics, including their location, size, and structure [9]. However, the complex and heterogeneous nature of brain tumors makes manual interpretation of MRI images challenging [10]. CNN offer a promising solution by automating the extraction of relevant features from visual data, thereby assisting clinicians in brain tumor classification and diagnosis.

Designing an optimal CNN architecture for MRI-based brain tumor classification presents substantial challenges [11]. This task requires an optimal configuration across a large parameter space that includes network depth, filter counts, convolutional layer dimensions, and numerous other hyperparameters. Designing these architectures manually to achieve peak performance is often labor-intensive and can lead to suboptimal outcomes. To streamline this process, evolutionary algorithms (EA) [12,13] have been employed. By simulating natural selection, EA generate a population of candidate solutions and improves them over successive generations using mechanisms like selection, crossover, and mutation [14]. EA have proven effective across various complex optimization problems, given their capacity to navigate large search spaces and yield high-quality solutions. A widely used EA is the Particle Swarm Optimization (PSO) which models collective behaviors observed in bird flocks or fish schools. In the context of CNN design, PSO facilitates the efficient, iterative search for an optimal architecture by treating each particle as a candidate solution. These particles refine their positions by balancing their individual learning with insights from their neighbors, progressively converging toward an optimal design [11,15,16]. Besides PSO, other evolutionary techniques, such as genetic algorithms (GA), have been utilized to optimize CNN architectures [17,18]. Furthermore, PSO has been applied to enhance medical image segmentation accuracy in brain tumors and lung CT scans, achieving notable improvements in Dice and Jaccard indices [19,20,21].

The present study proposed enhancements to the psoCNN algorithm, introduced in [22], to optimize CNN architectures for MRI-based brain tumor classification. By combining CNN’s feature extraction abilities with PSO’s optimization approach, the model aimed to achieve reliable diagnostic accuracy.

The key contributions of this work are the following:

- An initialization strategy is developed to predominantly configure particles with convolutional and pooling layers, ensuring that pooling layers are implicitly positioned after each convolutional layer.
- The search space is refined to focus on determining the optimal number of convolutional layers, their kernel sizes, as well as the ideal number of fully connected layers and their respective neuron counts.
- Incremental training is applied, allowing particles to undergo progressively deeper learning over time. This balances computational cost and performance while ensuring thorough evaluation of the best models.

This paper is divided into six sections. The related work section reviews prior studies and presents gaps addressed by this research. The Convolutional Neural Networks (CNN) section introduces the concepts of these algorithms outlining their structure and challenges. The Particle Swarm Optimization (PSO) section explains the optimization algorithm and its relevance to the problematic at hand. The application of PSO to CNN optimization section describes the proposed approach and methodology. The experimental results section presents findings and analyzes the effectiveness of the method. Finally, the conclusion summarizes the study’s contributions and suggests future research directions.

## 2. Related Work

Recent advancements in brain tumor classification using MRI have explored a range of methods, including manually designed CNN architectures, state-of-the-art pre-trained models, and approaches based on evolutionary algorithms. For instance, one study employed a genetic algorithm to evolve CNN architectures tailored to identify different glioma grades, achieving 90.9% accuracy in one case study and 94.2% accuracy in distinguishing between glioma, meningioma, and pituitary tumors [23]. However, the method faced challenges due to its expansive search space, which resulted in complex models. Another approach integrated a novel CNN model for feature extraction with classical machine learning algorithms, using Bayesian optimization to fine-tune hyperparameters [24]. This hybrid model outperformed nine state-of-the-art CNN models, achieving an impressive mean classification accuracy of 97.15%. Similarly, a different study combined a CNN with an SVM classifier and tested it on two datasets, yielding 99% accuracy for binary classification and 98% for multi-classification [25]. In a comparative analysis [26], a generic CNN model and six pre-trained models were evaluated with various preprocessing techniques. Among these, InceptionV3 stood out as the most accurate, achieving an average accuracy of 97.12%, surpassing other models. Another study proposed an automated approach for brain tumor classification using T1-weighted CE-MRI images, employing Bayesian optimization to optimize CNN hyperparameters. The model achieved an impressive 98.70% validation accuracy, surpassing well-known pre-trained architectures [27]. However, these custom and state-of-the-art models raise questions about the optimality of their architectures. Additionally, they may be overly complex for MRI-based brain tumor classification, particularly when using pre-trained models designed for broader applications. The present study focuses on identifying the optimal CNN architecture, ensuring it is well-suited for the task while striking a balance between model complexity and performance.

## 3. Convolutional Neural Networks (CNN)

**Figure 1:**
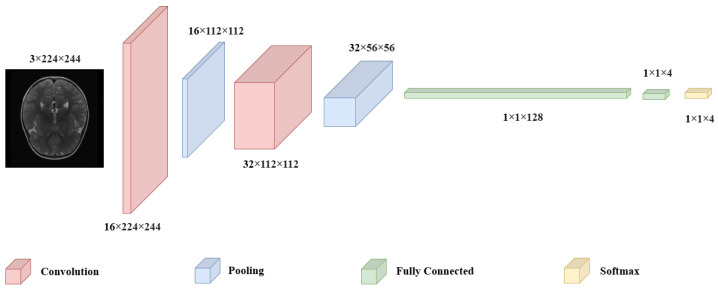
Architecture of a CNN.

CNN have emerged as indispensable tool in the field of medical imaging, owing to their exceptional ability to identify intricate patterns, particularly in brain tumor classification tasks. The substantial applicability of these models, demonstrating their superiority over alternative methods and highlight their potential to significantly improve diagnostic accuracy [28]. CNN utilize a layered approach, where convolutional layers extract spatial features by applying filters. Pooling layers reduce dimensionality while retaining essential information, and fully connected layers at the end aggregate these features for classification. In this design, convolution and pooling operations sequentially capture intricate image details, which the fully connected layers at the model’s tail use to make final predictions (Figure 1).

A CNN consists of interconnected neurons, each with specific weights and biases. These neurons take inputs from prior layers and perform calculations that combine the input values with the respective weights. CNN are designed with the assumption that the input data are images, which allows the model architecture to incorporate certain image-related features. The main types of layers within CNN include convolutional (C), pooling (P), and fully-connected (FC) layers [29]. These layers are organized sequentially, such as each layer’s output serves as the input for the next. Mathematically, a CNN can be defined as follows:

(1) $$\left\{\begin{array}{cc} O_{j}=X & \mathrm{if}\;j=1\\ O_{j}=f_{j}\left(Z_{j}\right) & \mathrm{if}\;j>1\\ Z_{j}=g_{j}\left(O_{j-1},W_{j}\right) \end{array}\right.$$

Here, *X* represents the input image, which can be represented as a tensor encoding the color channels and spatial size of the image, $f_{j}\left(\cdot \right)$ is the activation function at the *j*-th layer, $g_{j}\left(\cdot \right)$ represents the operation using weights at the *j*-th layer, $Z_{j}$ is the result from applying weights before activation, $W_{j}$ denotes the weights at the *j*-th layer, and $O_{j}$ is the output of the *j*-th layer.

### 3.1. Convolutional Layer (C)

**Figure 2:**
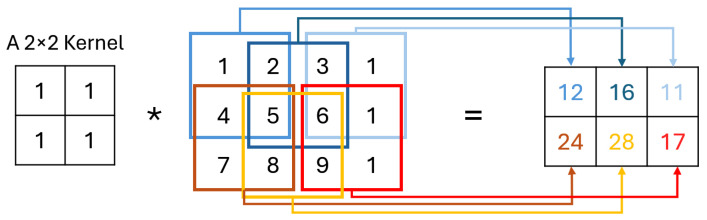
Convolution operation.

The C layer operates using small learnable filters which extend through the entire depth of the input but are narrow in spatial dimensions. As these filters traverse the input, they generate activation maps by calculating scalar products at each position, capturing important features [30], as shown in Figure 2. These activation maps, unique to each filter, stack together to form the output volume, allowing the network to learn specific spatial features. By training, CNN develop filters that detect patterns within localized areas, known as receptive fields, connecting only to limited input regions. Key hyperparameters—“number of filters”, “stride”, and “padding”—help control the model’s complexity and output dimensions. Adjusting stride, for instance, impacts the receptive field overlap, with smaller strides increasing overlap and larger strides reducing it, thereby affecting the spatial resolution of activations. Similarly, zero-padding around the input’s borders provides additional control over the output dimensions, enabling CNN to achieve greater flexibility in capturing spatial patterns [31].

### 3.2. Pooling Layer (P)

Pooling layers [32], reduce the spatial dimensions of feature maps, lowering computational demands and helping to prevent overfitting by downsampling. By transforming input data into a condensed representation, pooling layers focus on essential features while discarding less relevant information, thus reducing memory and computation requirements. Two main pooling types, local and global, offer distinct benefits: local pooling captures details within small regions, whereas global pooling compresses information into a scalar that summarizes features over the entire feature map. Among popular techniques, max pooling selects the maximum value within a region, preserving sharp and prominent features, while average pooling smooths the data by computing an average, capturing broader patterns but sometimes losing contrast. These pooling methods have core hyperparameters, such as those associated with the C layer.

### 3.3. Fully Connected Layer (FC)

FC layers play a critical role in combining the features extracted from C and P layers, transforming them into a final output suitable for classification or regression tasks. These layers are typically positioned towards the end of the network, where each neuron is connected to every neuron in the preceding layer, forming dense connections. This arrangement allows FC layers to capture complex relationships among features, but it also significantly increases the number of parameters, potentially leading to high computational costs and a tendency to overfit on small datasets. The number of neurons in each layer is a crucial hyperparameter in tuning fully connected layers, as it directly influences the model’s capacity to learn complex patterns.

### 3.4. Activation Function

To fully unlock the representational power of the previous layers, activation functions are introduced between them. Without activation functions, these layers would only perform linear transformations, limiting the network’s ability to capture intricate relationships in the data. They add the necessary nonlinearity, allowing the network to model more sophisticated patterns and interactions. Placed after each layer, they transform the output before it passes to the next, which directly impacts how well the network learns from data.

Historically, functions like Sigmoid and Tanh were commonly used; however, they often led to vanishing gradients in deep networks. This limitation prompted the introduction of ReLU, defined as f(x)=max(0,x), a simple yet powerful function that addresses the vanishing gradient issue for positive values, while requiring minimal computation. To address ReLU’s limitations, particularly for negative input values, researchers have developed several variations, including Leaky ReLU, PReLU, and other advanced functions, each designed to handle gradient issues more effectively and to improve overall network performance [33].

### 3.5. Softmax Cross-Entropy Loss

The softmax cross-entropy loss function is central to image classification, especially when handling multiclass problems [34]. As CNN generate predictions over multiple classes, this loss function quantifies the difference between the predicted probability distribution and the true distribution of the classes. It operates by applying the softmax function to convert output logits into probabilities across multiple classes, with the loss calculated based on the negative log likelihood of the correct class. By doing so, it ensures that each output probability lies between 0 and 1 and that the total sums to 1. Mathematically, if we denote the true label vector by y and the predicted probabilities by p, the cross-entropy loss L is given by:

(2) $$L=-\sum_{i=1}^{C}y_{i}\log\left(p_{i}\right)$$

where C represents the total number of classes, $y_{i}$ is 1 for the correct class and 0 otherwise, and $p_{i}$ is the predicted probability for class i.

### 3.6. Training CNN

Optimizing CNN architectures requires careful integration of effective layer configurations, regularization strategies, and efficient training processes to create models that perform well and generalize effectively. Regularization is a key component in this optimization process, addressing the challenge of overfitting and improving model robustness [35]. Techniques like batch normalization stabilize the training process by normalizing the inputs within each layer, which minimizes the internal covariate shift that often slows down training. By maintaining a consistent input distribution, batch normalization allows for faster and more stable convergence and supports the use of higher learning rates, further accelerating the learning process. Dropout, another important regularization technique, reduces overfitting by randomly deactivating a percentage of neurons during each training iteration. This discourages the network from depending heavily on specific neurons, encouraging it to develop a more distributed, resilient representation of the data, especially within FC layers. Training CNN effectively also requires robust optimization algorithms alongside backpropagation which the processs of calculating the gradient of the loss function relative to each parameter. Optimizers such as Stochastic Gradient Descent (SGD) and adaptive methods like Adam and RMSprop are essential for efficiently updating weights and achieving convergence, as they balance learning rate adjustments and momentum to navigate complex parameter spaces effectively [36].

## 4. Particle Swarm Optimization

PSO is a nature-inspired, population-based algorithm that simulates the collective behavior observed in groups of animals, such as flocks of birds, schools of fish, and insect swarms. Introduced by Eberhart and Kennedy in 1995 [37], PSO models how these groups coordinate to find resources, with each individual adjusting its movement by learning from both its own experience and the collective insights of the swarm. The algorithm has since evolved to address a wide range of complex optimization challenges. Researchers have developed numerous PSO variants and adaptations, targeting specific application needs and exploring different parameter settings, topology configurations, and multi-objective capabilities [38]. This algorithm remains popular in engineering, machine learning, and other fields for its adaptability, efficiency in parallel computing, and quick convergence to optimal solutions. However, current research tends to emphasize application and enhancement, with foundational theoretical studies lacking, restricting its full potential.

In PSO, each particle within the swarm represents a possible solution, with its movement through the solution space governed by iterative updates to both its position and velocity. The particle’s velocity at each iteration reflects a balance between the particle’s own historical experience and the collective experience of the swarm. The velocity update involves adjusting how fast and in which direction the particle is moving, and it is calculated by combining its current velocity with adjustments based on two factors: the particle’s best-known position, referred to as the personal best (pBest), and the best-known position found by any particle in the swarm, known as the global best (gBest). This velocity update can be expressed mathematically as:

(3) $$v_{i}\left(t+1\right)=\omega v_{i}\left(t\right)+c_{1}r_{1}\left(pBest_{i}-x_{i}\left(t\right)\right)+c_{2}r_{2}\left(gBest-x_{i}\left(t\right)\right)$$

where ω represents the inertia weight, which determines the influence of the particle’s prior velocity, encouraging continuity in its motion. The parameters $c_{1}$ and $c_{2}$, often called the cognitive and social coefficients, control the particle’s tendency to be guided by its own previous successes (cognitive factor) or by the success of the swarm as a whole (social factor). Random variables $r_{1}$ and $r_{2}$, uniformly distributed between 0 and 1, introduce stochasticity, allowing for a broader exploration of the solution space. After updating the velocity, the particle moves to a new position by adding this velocity to its current position:

(4) $$x_{i}\left(t+1\right)=x_{i}\left(t\right)+v_{i}\left(t+1\right)$$

This process continues iteratively, with particles refining their search paths based on ongoing updates from both individual and collective feedback. This cycle repeats until a stopping condition, such as reaching an optimal solution threshold or achieving a predefined number of iterations, is satisfied.

## 5. Application of PSO to the Optimization of CNN Architecture

**Figure 3:**
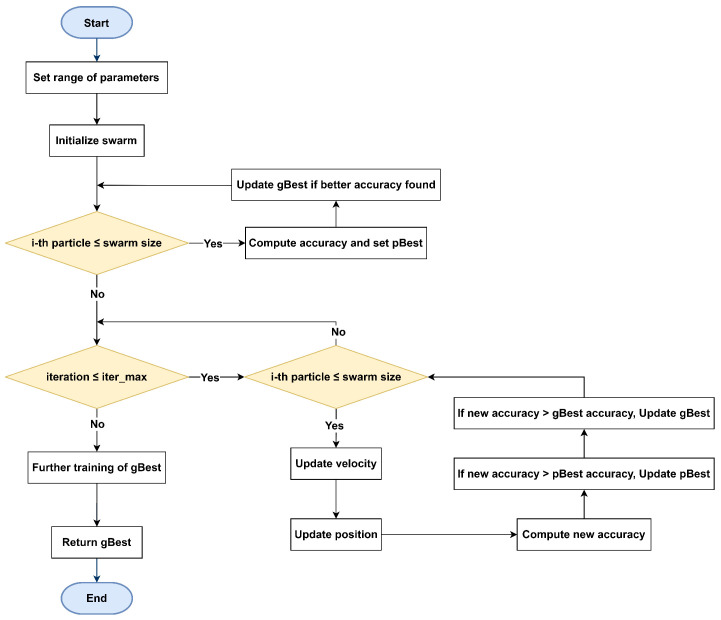
Flowchart of the algorithm starting with parameter initialization, swarm creation, and fitness evaluation. Each particle iteratively updates its position and velocity using pBest and gBest, until the maximum number of iterations is reached.

The core structure of the proposed psoCNN algorithm is illustrated in Figure 3. This framework accepts input data related to the optimization and classification task, including the training dataset and hyperparameters for CNN architecture generation, such as the maximum allowable number of layers at initialization. The algorithm determines the global best particle (gBest) by using PSO to select the most effective layers within the swarm, eliminating the need for manual fine-tuning of each layer’s hyperparameters. Through this approach, high-quality layers from prior generations are retained in the optimization process, rather than being reinitialized with every iteration. Although particles undergo reassessment during each iteration, well-performing layers are preserved, enabling valuable features to carry over from one generation to the next.

This algorithm adheres to the PSO structure and consists of six core steps: efficient CNN encoding, initialization of the swarm, assessment of each particle’s fitness, evaluation of differences between particles, computation of velocities, and particles’ update. The following sub-sections describe these steps in more detail, emphasizing the initialization and fitness evaluation improvements that contribute to enhanced performance.

### 5.1. Particle-Based Encoding Scheme

**Figure 4:**
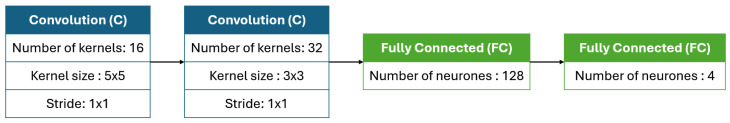
An encoded CNN architecture with two convolutional layers: the first with 16 kernels of size 5 × 5 and the second with 32 kernels of size 3 × 3, each followed by 2 × 2 max pooling. The architecture ends with two fully connected layers containing 128 and 4 neurons.

One of the key challenges in adapting a PSO algorithm to identify optimal CNN architectures lies in designing an encoding strategy, which involves representing CNN architectures in a structured and quantifiable format. This structured representation allows the algorithm to interpret the particle’s position and facilitates effective velocity updates during the optimization process. In this encoding format, each particle directly represents CNN layers without the need for numerical transformation, using a straightforward structure. The encoding is organized as a list of lists, where each sub-list corresponds to one specific layer, with details on its hyperparameters. In this framework, convolutional and max pooling layers are integrated, treated as a single combined operation rather than independent layers. Each convolutional layer (C) is paired with a max pooling operation, consistently using a kernel size of 2 × 2 and a stride of 2 × 2. This ensures a simplified and uniform structure for layer encoding. The attributes for convolutional layers include the number of filters, filter size, and stride, while the pooling operation is implicitly applied as part of the convolutional layer. Fully connected (FC) layers are also represented in the encoding, specifying the number of neurons. As illustrated in Figure 4, this encoding scheme constructs the CNN model by interpreting each particle component sequentially from left to right, adding each layer as specified. Importantly, this encoding does not store weight values, hence the incorporation of a brief retraining phase to compute the accuracy of each particle.

### 5.2. Swarm Initialization

The swarm initialization process begins by generating N particles, where each particle represents a CNN architecture with a randomly configured set of layers. The architectures can have between three and a maximum number of layers, with the first layer always set to a C layer and the final layer to a FC layer. To preserve the structural integrity of the CNN, FC layers are restricted to the end of the architecture, avoiding their placement between C layers. The C layers are initialized with randomly selected numbers of kernels and kernel sizes, and every C layer is immediately followed by a max pooling layer with predefined window sizes and stride values. The FC layers at the end of the network are assigned a random number of neurons. All layers employ the ReLU as the activation function. To ensure functional CNN architectures, the process also manages the placement of pooling layers to avoid reducing the output dimensions below 7 × 7. Additionally, particles are initialized with a balanced structure, where approximately two-thirds of the layers are either convolutional or pooling layers, and one-third are fully connected layers, allowing for both effective feature extraction and classification capabilities.

### 5.3. Fitness Evaluation

Fitness evaluation refers to the process of assessing the performance of each particle by converting it into a CNN model and training it for an initial number of epochs. The accuracy of each CNN is then evaluated on a validation set, aiming to identify the architecture with the highest performance. This process utilizes the Adam optimizer for efficient convergence, applies a dropout rate of 20% just before the final FC layer to prevent overfitting. Weight initialization follows the standard configuration in PyTorch [39]. However, a significant bottleneck arises as every particle must be trained on the full dataset, making this evaluation phase time intensive. The algorithm is designed specifically to discover the most effective CNN architecture rather than focusing on fine-tuning its weights. Initially, each particle undergoes training for a preset number of epochs, and with each iteration, the training duration is increased by one epoch to refine accuracy measurements. For a comprehensive evaluation of the gBest obtained, a final retraining phase is conducted using an extended number of epochs to solidify the performance assessment.

### 5.4. Computation of Difference Between Particles

**Figure 5:**
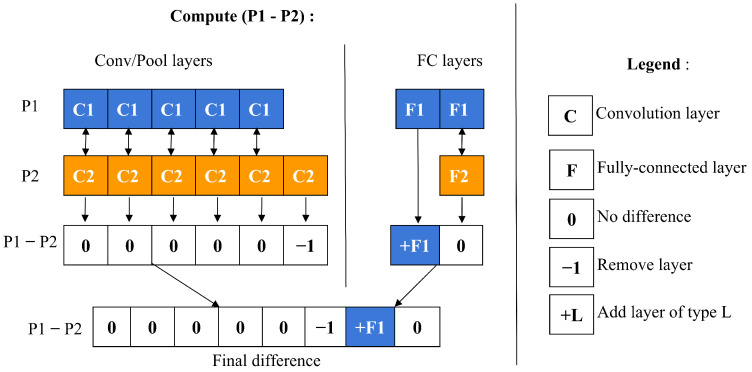
Calculation of the difference between two particles.

**Figure 6:**
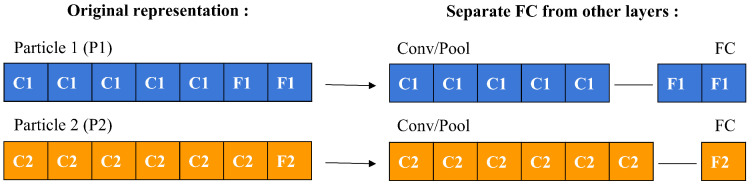
Separating FC layers from other layers.

To compute a particle’s velocity and subsequently update its position, a specific operator is employed to measure the symbolic difference between two particles. This process, depicted in Figure 5, involves a detailed comparison between particles labeled as P1 and P2. Initially, the layers of each particle are separated into two categories: convolutional/pooling (C/P) layers and fully connected (FC) layers, as demonstrated in Figure 6.

These two categories of layers are then assessed individually to identify any structural differences. The comparison is made relative to P1 by examining the C/P and FC layer groups independently. For C/P layers, differences are assessed from left to right, whereas for FC layers, the comparison proceeds from right to left. If the layer types match between P1 and P2, the difference is zero. If they differ, the difference is determined by P1’s layer type. When P1 contains fewer layers than P2, a difference of −1 is assigned, suggesting the removal of a layer from P2. Conversely, if P1 has more layers, a difference of +L is indicated, where L represents P1’s layer type, suggesting the addition of a layer to P2.

### 5.5. Particle Velocity Computation

**Figure 7:**
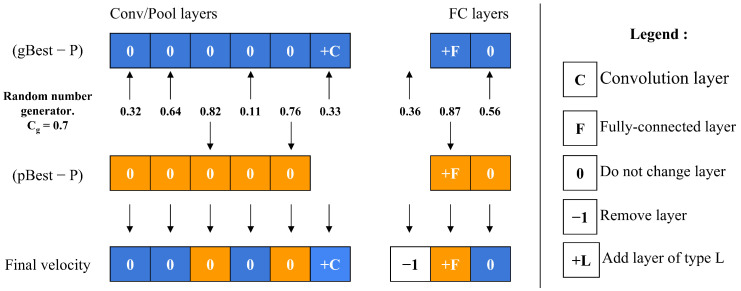
Velocity calculation of a single particle.

**Figure 8:**
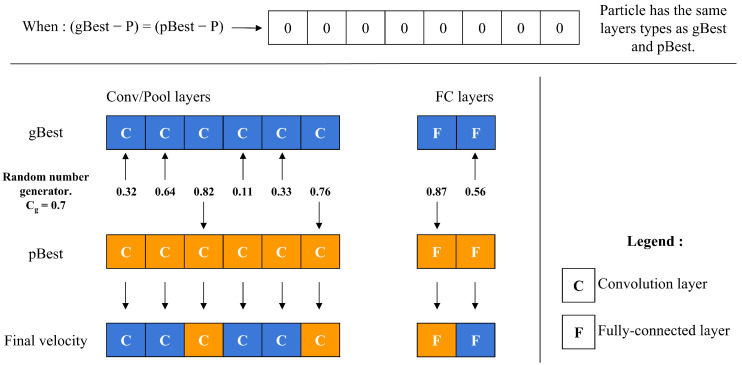
Particle velocity calculation when gBest and pBest are the same.

The velocity operator calculates two main differences: (gBest-P) and (pBest-P). To decide which difference to apply to each layer, the operator employs a threshold value, $C_{g}$ along with a random number generator. For each layer, it compares the random number to $C_{g}$; if the number is below $C_{g}$, the layer difference from (gBest-P) is chosen, whereas if the number is above $C_{g}$, the layer difference from (pBest-P) is selected instead, as depicted in Figure 7. This decision process repeats for every layer, allowing $C_{g}$ to control the particle’s resemblance to either gBest or pBest. As $C_{g}$ approaches 1, the particle’s structure aligns more closely with gBest. A unique scenario arises during the final iterations if (gBest-P) equals (pBest-P). In this case, the operator decides between adopting gBest or pBest directly, based on the value of $C_{g}$, as shown in Figure 8.

### 5.6. Particle Position Adjustment

**Figure 9:**
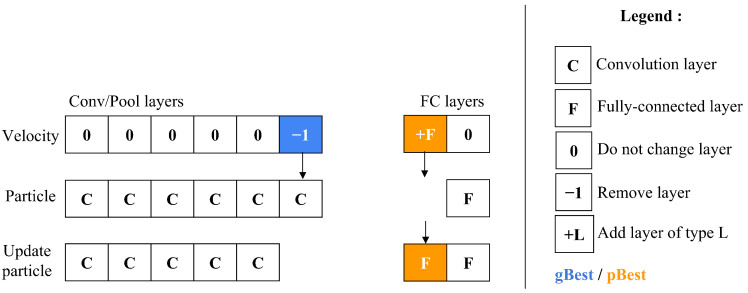
Updating the architecture of a particle.

After calculating a particle’s velocity, the update particle operator is then applied to modify the particle’s configuration. This adjustment process, shown in Figure 9 entails a comparison between the particle’s current velocity and its existing position. The operator handles the C/P and FC layer blocks individually, ensuring that updates are made only to the position components where velocity is non-zero. Through this mechanism, particles have the flexibility to evolve over time—either contracting by removing layers or expanding by adding layers to the particle’s architectural structure.

## 6. Experimental Results

### 6.1. Dataset

**Figure 10:**
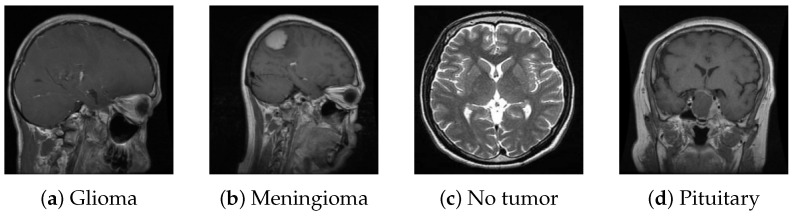
Samples from the dataset.

**Figure 11:**
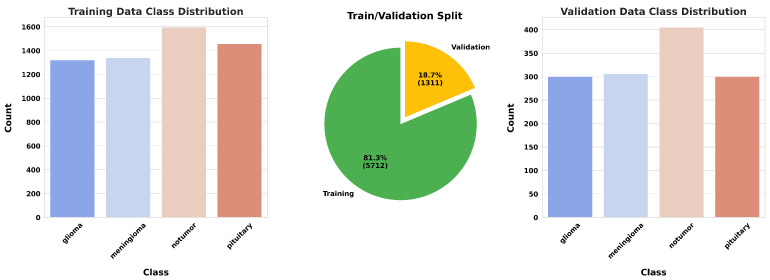
Data distribution.

For this study, we utilized a publicly available MRI dataset from Kaggle [40], comprising 7023 brain MRI images sorted into four categories: glioma, meningioma, pituitary, and no tumor. This dataset integrates images from several sources, including Figshare [41], the SARTAJ dataset [42], and Br35H [43], with non-tumor images primarily sourced from Br35H. Figure 10 presents sample images from each class, while Figure 11 illustrates the distribution of these images across each category.

### 6.2. Algorithm Parameters

The parameters utilized in this study fall into three main categories: those associated with PSO configuration, those related to the initialization of CNN architectures and those governing the evaluation process for individual particles. The parameters specific to the PSO process, summarized in Table 1, define essential aspects such as the termination criteria, the size of the swarm, and the rate ($C_{g}$) at which particles converge towards the global best (gBest). A larger swarm or increased iteration count improves the probability of achieving optimal solutions, albeit at a higher computational cost.

The CNN initialization parameters are presented in Table 2. These parameters specify the range of configurations possible for the CNN architectures generated within the swarm. Each particle’s architecture is initialized by randomly selecting values within these predefined limits.

Finally, the parameters for particle evaluation and final best particle training are detailed in Table 3. These parameters include the starting number of epochs used during the evaluation phase of individual particles and the extended training of the best-performing architecture after optimization concludes.

### 6.3. Results

**Figure 12:**
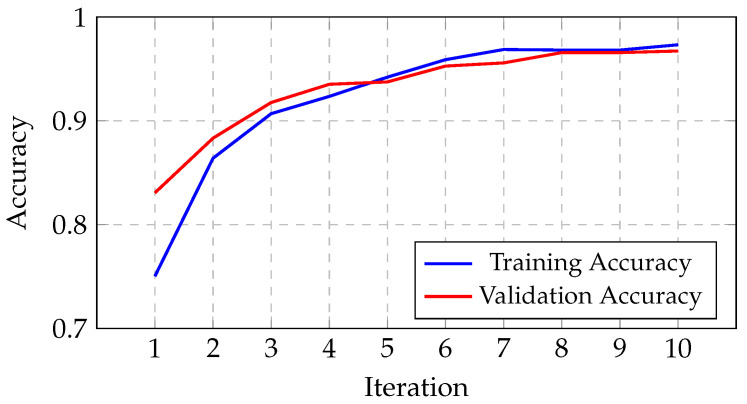
Progression of the gBest model’s accuracy through iterations.

The algorithm’s results are presented in this sub-section, highlighting the progression of the gBest model’s accuracy over iterations. As shown in Figure 12, the training accuracy increases from 75.04% in the initial iteration to 97.32% by the tenth iteration. Similarly, the validation accuracy improves from 83.07% to 96.72% over the same period. This progression reflects the algorithm’s effectiveness in exploring and optimizing architectural configurations. The architecture founded by the algorithm, detailed in Table 4, utilizes a first convolutional layer with 5 × 5 kernels and a second layer with 3 × 3 kernels. This architecture comprises a total of 12,851,556 trainable parameters, enabling effective feature extraction and classification. The training process was carried out using a mini-batch of 32 images.

**Figure 13:**
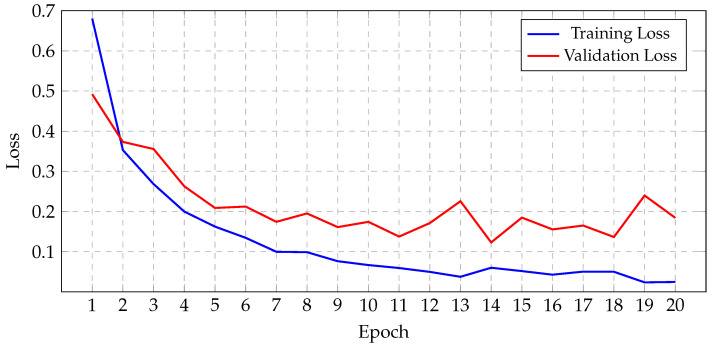
Progression of gBest training and validation loss.

**Figure 14:**
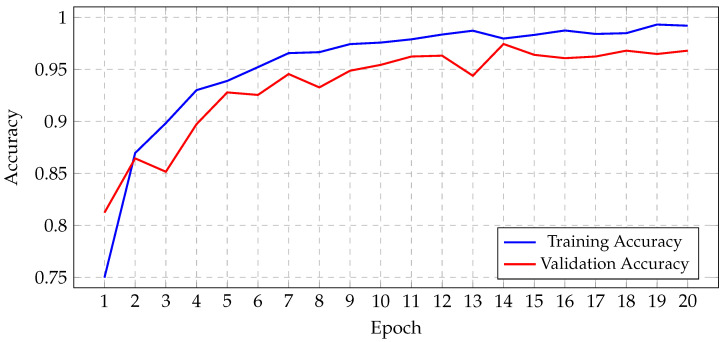
Progression of gBest training and validation accuracy.

Before initiating the training of the final gBest particle, 5% of the dataset was set aside for testing and prediction purposes. This testing set was created by sampling 81.3% of the 5% from the training data and 18.7% from the validation data. This procedure ensured that the class distribution in the testing subset aligned with the original proportions of the training and validation data. The gBest particle model completed its training within 20 epochs. The training process resulted in a steady reduction in training loss, starting from 0.68 and decreasing to 0.024 by the final epoch. Correspondingly, the training accuracy improved from 74.97% at the beginning to 99.18% at the end. The validation loss exhibited an initial value of 0.492, fluctuating throughout the epochs, and concluded at 0.184. Similarly, the validation accuracy increased from 81.2% to 96.8%. These trends are illustrated in Figure 13, which shows the progression of loss over the epochs, and Figure 14, which displays the accuracy trends for both training and validation data.

**Figure 15:**
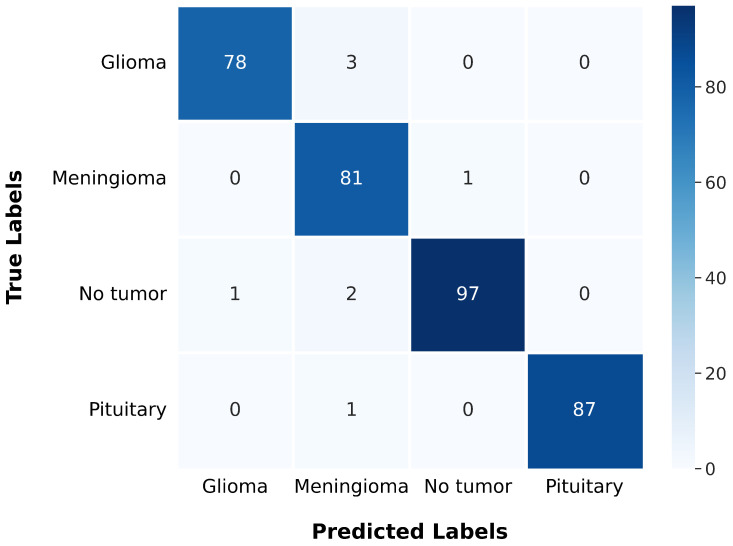
The gBest confusion matrix.

After training, the model was evaluated using the test data, yielding a test loss of 0.137 and a test accuracy of 97.72%. The model’s classification performance was further analyzed using a confusion matrix, presented in Figure 15. The confusion matrix offers a comprehensive understanding of a model’s classification performance by illustrating the correspondence between actual and predicted labels. It emphasizes essential components of classification results and serves as the basis for evaluating metrics such as precision, recall, F1 score, and Matthews Correlation Coefficient (MCC), which are summarized in Table 5 for each class.

### 6.4. Discussion

The gBest confusion matrix provides a detailed insight into the classification of different tumor types. For Glioma, the model correctly identified 78 cases, with 3 false negatives and 1 false positive, suggesting a strong ability to detect this type of tumor. Meningioma classification was slightly less precise, with 81 correct identifications, 1 false negative, and 6 false positives, indicating a minor trade-off in precision. The model excelled in identifying No Tumor cases, with 97 correct classifications, only 3 false negatives, and 1 false positive, reflecting its high reliability in this category. Pituitary tumor classification was nearly flawless, with 87 correct identifications and only 1 false negative.

The model’s performance metrics, including precision, recall, and F1 scores, demonstrate its robust capabilities. The Glioma classification exhibits high precision and recall, signifying a low rate of errors in identifying this tumor type. The Meningioma classification shows a high recall but slightly lower precision, suggesting the model effectively identifies most Meningiomas while producing a few additional false positives. The No Tumor classification boasts near-flawless precision and recall, underscoring the model’s robust ability to accurately detect the absence of tumors. The Pituitary tumor classification is virtually perfect, with 100% precision and high recall, indicating exceptional accuracy in this specific category. These results underscore the success of using PSO in searching for the optimal architecture, leading to a highly successful and generalizable model. This method proved to be efficient in identifying a well-suited configuration, thereby yielding acceptable performance in our context. The integration of automated optimization techniques not only streamlines the architecture selection process but also ensures that the model is tailored effectively to the task at hand.

Table 6 provides a comparative performance analysis of the proposed model and a similar model that employed a Genetic Algorithm for MRI brain tumor classification [23]. The proposed method consistently achieves higher accuracy across all tumor types. For glioma classification, the proposed approach achieves an accuracy outperforming the GA-based approach (Table 6). Similarly, the accuracy for meningioma classification improves in the proposed approach. The most significant improvement is observed in pituitary tumor classification, where the proposed method exceeded the GA-based approach.

In addition to these performance improvements, independent trials and observations on the dataset revealed that the characteristics of MRI brain tumor images do not require overly complex models for effective classification. This insight prompted a reduction in the range of hyperparameters explored during the algorithm optimization process. By focusing on identifying simpler models, the proposed method was able to achieve a balance between complexity and performance, resulting in a model that not only outperformed the GA-based approach in terms of accuracy but also offered a streamlined design. As shown in Table 7, our proposed CNN achieved the highest validation accuracy with a moderate number of parameters, outperforming more complex models. This indicates that leveraging PSO for architecture optimization is an effective strategy for identifying optimal solutions with reduced complexity, particularly in cases where the image data does not demand intricate models for accurate classification.

**Figure 16:**
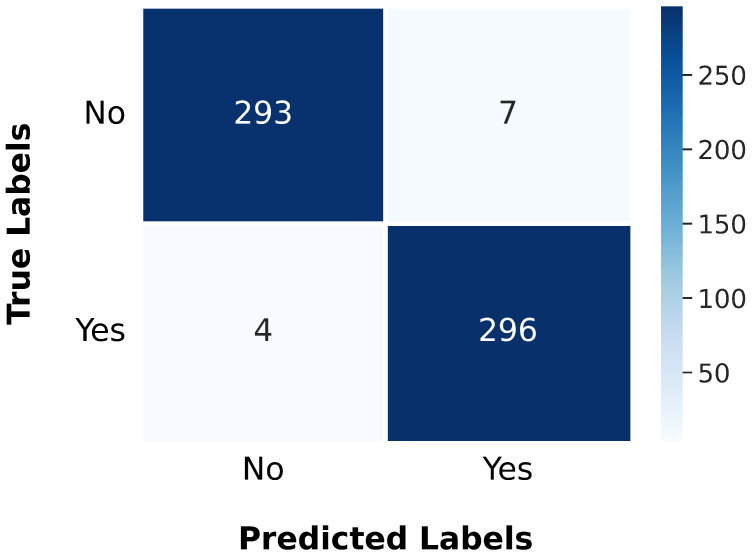
Confusion matrix illustrating the gBest model’s classification accuracy and error distribution on the BTD-MRI test set.

To assess the performance of the gBest model, we extended our experiments to the BTD-MRI dataset, which is publicly available on Kaggle [44]. This dataset comprises two subsets: one containing 1500 MRI images labeled as “no” (indicating the absence of tumors) and the other containing 1500 images labeled as “yes” (indicating the presence of tumors). We allocated 80% of the dataset for training and reserved the remaining 20% for testing. The model was trained using a batch size of 64, a dropout rate of 0.25, and 20 epochs. During training, the model achieved a loss of 0.0156 and an accuracy of 99.62%. When evaluated on the test set, it recorded a loss of 0.0839 and an accuracy of 98.17%. The performance results are further illustrated by the confusion matrix shown in Figure 16.

Table 8 presents a comparison of various techniques used for the classification of MRI brain tumor images. It details the methods employed, the datasets used, the number of classification categories, the top-performing models, and their validation accuracies. The techniques include methods such as genetic algorithms, state-of-the-art CNN models optimized with machine learning techniques, and CNN Bayesian hyperparameter optimization. Each method shows significant performance, with accuracies that vary depending on the dataset and the complexity of the classification task. However, it is important to note that the gBest model identified by the proposed algorithm represents an initial step in optimizing the CNN architecture. Further refinement and optimization would be necessary to improve the model’s performance and provide a more comprehensive basis for comparison with other methods.

## 7. Conclusions

The main findings of the present study indicate that PSO can successfully automate the CNN design process, achieving improved performance in classifying MRI images of brain tumors. These results suggest that PSO has potential applications in optimizing CNN architectures for various medical imaging tasks.

Nevertheless, the approach has certain limitations. The used algorithm relies on hyperparameters of the initialized architectures, which may restrict the exploration of alternative solutions. Addressing this limitation by incorporating mechanisms to adjust hyperparameters dynamically during optimization and adhering to established architectural conventions during initialization may improve the algorithm’s flexibility and performance.

Future research could build upon this study by incorporating additional computational resources to facilitate broader evaluations and testing on diverse datasets and tumor types to assess generalizability. Investigating hybrid optimization techniques that combine PSO with other algorithms could further enhance performance. Additionally, extending the PSO framework to optimize other neural network types, such as Recurrent Neural Networks (RNNs) or Long Short-Term Memory (LSTM) networks, may provide insights into its applicability beyond CNN architectures.

## Figures and Tables

**Table 1:** PSO Parameters.

Description	Value
Number of iterations	10
Swarm size	15
$C_{g}$	0.5

**Table 2:** CNN Architecture Initialization Parameters.

Description	Value
Max number of filters	40
Max filter size	7×7
Max neurons in FC layer	140
Max number of layers	9

**Table 3:** Particle Training Parameters.

Description	Value
Starting epochs for particle evaluation	1
Epochs for final best particle training	40

**Table 4:** The optimal CNN architecture identified through the algorithm.

Layer (Type)	Output Shape	Param #
ZeroPad2d-1	[32, 3, 228, 228]	0
Conv2d-2	[32, 16, 224, 224]	1216
ReLU-3	[32, 16, 224, 224]	0
MaxPool2d-4	[32, 16, 112, 112]	0
ZeroPad2d-5	[32, 16, 114, 114]	0
Conv2d-6	[32, 32, 112, 112]	4640
ReLU-7	[32, 32, 112, 112]	0
MaxPool2d-8	[32, 32, 56, 56]	0
Linear-9	[32, 128]	12,845,184
ReLU-10	[32, 128]	0
Dropout-11	[32, 128]	0
Linear-12	[32, 4]	516

Total params: 12,851,556. Estimated Total Size (MB): 817.83

**Table 5:** Evaluation metrics calculated from the confusion matrix for each class.

Class	TP	FP	FN	TN	Precision	Recall	F1 Score	Accuracy	MCC
Glioma	78	1	3	269	0.9873	0.9629	0.9750	0.9886	0.9677
Meningioma	81	6	1	263	0.9310	0.9878	0.9585	0.9800	0.9462
No Tumor	97	1	3	250	0.9897	0.9700	0.9798	0.9886	0.9719
Pituitary	87	0	1	263	1.0000	0.9886	0.9943	0.9971	0.9924

TP = True Positive; FP = False Positive; FN = False Negative; TN = True Negative Precision = $\frac{\mathrm{TP}}{\mathrm{TP}+\mathrm{FP}}$; Recall = $\frac{\mathrm{TP}}{\mathrm{TP}+\mathrm{FN}}$; F1 Score = $\frac{2\times \mathrm{Precision}\times \mathrm{Recall}}{\mathrm{Precision}+\mathrm{Recall}}$; Accuracy = $\frac{\mathrm{TP}+\mathrm{TN}}{\mathrm{TP}+\mathrm{TN}+\mathrm{FP}+\mathrm{FN}}$ Matthews Correlation Coefficient (MCC) = $\frac{\mathrm{TP}\times \mathrm{TN}-\mathrm{FP}\times \mathrm{FN}}{\sqrt{\left(\mathrm{TP}+\mathrm{FP})(\mathrm{TP}+\mathrm{FN})(\mathrm{TN}+\mathrm{FP})(\mathrm{TN}+\mathrm{FN}\right)}}$.

**Table 6:** Comparison of Classification Accuracy: PSO-Optimized versus GA-Optimized Approaches.

Approach	Class	Accuracy
GA + CNN [23]	Glioma	0.9652
	Meningioma	0.9449
	Pituitary	0.9739
PSO + CNN	Glioma	0.9886
	Meningioma	0.9800
	Pituitary	0.9971

**Table 7:** Comparison of the proposed CNN with other Models [24] based on number of parameters (Millions) and validation accuracy.

CNN Model	Number of Parameters	Validation Accuracy
DarkNet19	20.8	0.9295
DarkNet53	41.6	0.9380
DenseNet201	18.9	0.9316
EfficientNetB0	5.3	0.9551
InceptionV3	23.9	0.9174
NasNetMobile	4.4	0.9003
ResNet50	25.6	0.9509
ResNet101	44.6	0.9544
Xception	22.9	0.9224
Generic CNN	10.9	0.9566
Proposed CNN	12.85	**0.9680**

**Table 8:** Comparison of the proposed method with previous studies.

Reference	Method	Dataset	Classes	Best Model	Accuracy (%)
Ref. [23]	GA + CNN	Figshare [41]	3	CNN	94.2
Ref [24]	State of the art CNN-optimized ML	Combination [40]	4	EfficientNetB0-SVM	97.93
Ref. [27]	CNN HPO	Figshare [41]	3	CNN	98.70
Ref. [26]	CNN with TL	Combination [40]	4	Inception V3	97.12
Ref. [45]	DL-based model	BTD-MRI [44]	2	TumorResNet	99.33
Ref. [46]	Spectral Data Augmentationbased Deep Autoencoder	BTD-MRI [44]	2	SDA-DA CNN	97
This work	Proposed Method	Combination [40]	4	gBest model	96.8
This work	Proposed Method	BTD-MRI [44]	2	gBest model	98.17

## Data Availability

The dataset used in this study, the “Brain Tumor MRI Dataset”, is publicly available on Kaggle and can be accessed at https://www.kaggle.com/dsv/2645886 (accessed on 13 December 2024).
